# Systematic review estimating the burden of dementia in the WHO Southeast Asia Region using Bayesian and frequentist approaches

**DOI:** 10.7189/jogh.10.020701

**Published:** 2020-12

**Authors:** Adrienne N Poon, Yawen Xiang, Yelena Zavalishina, Shant Ayanian, Christopher F Aitken, Andrew C Procter, Igor Rudan, Kit Yee Chan

**Affiliations:** 1 Centre for Global Health Research, Usher Institute of Population Health Sciences and Informatics, University of Edinburgh, UK; 2 Department of Medicine, School of Medicine & Health Sciences, George Washington University; Washington, District of Columbia, USA; 3 Department of Economics, School of Social Sciences, Heriot-Watt University, Edinburgh, UK; 4 Accenture Federal Services, Arlington, Virginia, USA; 5 Nossal Institute for Global Health, Melbourne School of Population and Global Health, University of Melbourne, Australia

## Abstract

**Background:**

Rapid increase in life expectancy in low- and middle-income countries including the World Health Organization’s Southeast Asia Region (SEAR) has resulted in an increase in the global burden of dementia, which is expected to become a leading cause of morbidity. Accurate burden estimates are key for informing policy and planning. Given the paucity of data, estimates were developed using both a Bayesian methodology and as well as a traditional frequentist approach to gain better insights into methodological approaches for disease burden estimates.

**Methods:**

Seven databases were searched for studies published between 2010-2018 regarding dementia prevalence in SEAR, generating 8 relevant articles. A random-effects model (REM) and a Bayesian normal-normal hierarchical model (NNHM) were used to obtain the pooled prevalence estimate of dementia for people aged 60 and above in SEAR. The latter model was also developed to estimate age-specific dementia prevalence. Using UN population estimates for SEAR, total and age-specific projections of the burden of dementia in 2015, 2020 and 2030 were calculated.

**Results:**

The prevalence of dementia in SEAR was found to be 3% (95% confidence interval (CI) = 2-6%) in those above age 60 based on REM, and 3.1% (95% credible interval = 1.5-5.0%) based on the NNHM. The estimated prevalence varies with age, increasing from 1.6% (95% credible interval = 0.8-2.5%) in people aged 60-69 to 12.4% (95% credible interval = 5.6-20%) in people above the age of 80. The risk of developing dementia increased exponentially with age. The number of people living with dementia in SEAR in 2015 was estimated at 5.51 million (95% credible interval = 2.66-8.82), with projections of 6.66 million (95% credible interval = 3.21-10.7) in 2020 and 9.6 million (95% credible interval = 4.62-15.36) in 2030.

**Conclusion:**

The burden of dementia in SEAR is substantial and will continue to increase rapidly by 2030. The lack of research focusing on dementia in SEAR points to a significant under-recognition of this disease. The projected rise in dementia cases in the future should prompt urgent governmental response to address this growing public health issue. We also argue that given the overall paucity of data for the region, the Bayesian approach offers a promising methodology for improved estimates of disease prevalence and burden and should continue to be explored.

In 2015, an estimated 47 million people worldwide suffered from dementia, a major incapacitating syndrome defined by the progressive loss of cognitive ability and independent living beyond normal aging [1]. This figure is predicted to rise to 75 million by 2030, and 132 million by 2050, and has a bigger economic impact than cancer, heart disease and stroke combined [2]. In 2015, an estimated US$818 billion was spent on dementia (1.1% of global Gross Domestic Product), and that is expected to rise to US$2 trillion by 2030 [1]. Due to their large, rapidly aging populations, low- and lower-middle-income countries (LMICs) are expected to bear an increasing majority of this burden, accounting for up to 71% of global dementia cases by 2050 [1]. The condition not only negatively affects the quality of life of people living with dementia (PWD), but also imposes significant financial, emotional and opportunity costs on their families and caregivers, and it strains social and health resources. Despite medical advancements and breakthroughs taking place in this century, there is currently no cure or disease-modifying treatment.

The World Health Organization’s (WHO) Southeast Asia Region (SEAR), consisting mainly of LMICs, is the second most populated region of the world, with a quarter of its population (1.9 billion) [3]. There have only been three prior estimates that include dementia in the SEAR region, with the more recent covering estimates for the period of 1980-2009 [4-6]. Rapid economic, health and demographic transitions have occurred in this region since the last estimates were published, leading to a rise of non-communicable diseases including dementia. The evidence for dementia in the SEAR region has also been expanded over the last decade. Therefore, a separate and more comprehensive study of SEAR with updated estimates is urgently needed.

While disease burden estimates have traditionally relied on the frequentist analytic approach [7-9], Bayesian methods are growing in popularity in medical research (e.g., Prince 2013 [5]; Nichols 2019 [6]. The latter framework is considered more appropriate for meta-analyses with very few available studies because it allows additional information to be incorporated into current estimates [7,8]. That additional information, embedded in a prior, introduces a natural form of regularisation into the estimation procedure, helping to improve precision. Other, similar meta-analyses are ideal sources for that information: they represent credible distillations of available scientific evidence. With existing meta-analytic evidence forming the basis for the prior, the posterior produced by the Bayesian procedure can be interpreted as an updated summary. This in turn ensures consistency between current and prior estimates [9]. Two global health organizations that work on the global prevalence of dementia have both opted for Bayesian methods. The estimates produced by Prince and colleagues in 2013 assumed that the prevalence of dementia follows a gamma distribution [5]. The latest regional dementia estimate published in 2019 by the Institute for Health Metrics and Evaluation (IHME) used a Bayesian method [6]. An optimal approach, however, has not yet been determined and methods for Bayesian application to prevalence estimates is an area of ongoing study.

The overall aim of this study is to update the dementia prevalence estimates for SEAR with greater accuracy through utilizing two approaches to estimation: frequentist and Bayesian. We aim to achieve this by conducting a comprehensive systematic review of data that has emerged from the region since 2009, when the previous estimates were published, using a greater number of academic databases. Given the paucity of data, we decided to use the Bayesian approach with confirmation by the traditional frequentist approach to generate more reliable estimates for disease burden. Generating appropriate models for Bayesian analysis may be challenging and thus we explored the use of the Bayesian Random-Effects Meta-Analysis (bayesmeta), a newer and simpler to operate statistical open source package in R. It is hoped that the updated estimates will help draw attention to the growing burden of dementia in SEAR as part of a global trend. This study can inform policy and planning, while methodological insights generated from the comparison of analytic approaches will guide development of future disease burden estimates.

## METHODS

### Study selection

We sought to include prospective population-based studies of dementia prevalence in countries in the WHO’s SEAR published between 2010 and November 2018. The study was conducted and reported in accordance with the Preferred Reporting Items for Systematic Reviews and Meta-Analyses (PRISMA) guidelines [10] and involved a parallel systematic review conducted in May and November 2018 of studies on the epidemiology of dementia. Seven databases were searched: PubMed, MEDLINE, EMBASE, Global Health Library (CABI), Global Index Medicus, PsycInfo, and the BIOSIS Citation Index (Table S1 and Appendix S1 in the **Online Supplementary Document**).

**Figure 1:**
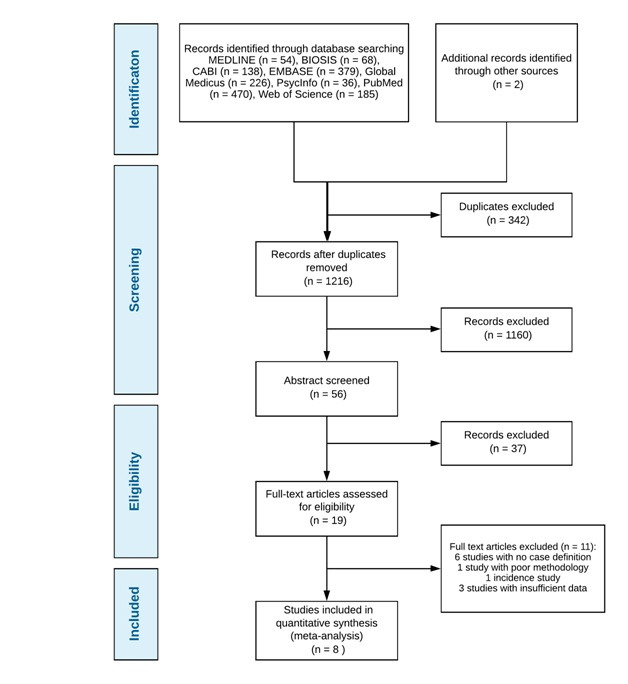
PRISMA flow diagram for selection of studies included in the systematic review.

1,556 studies were identified with 2 additional studies found through hand searches of reference lists. After the removal of duplicates, 1,216 articles remained of which 1,160 articles were further excluded based on the title relevance. 56 abstracts were then screened and 19 full–text articles were then analysed for inclusion/exclusion criteria, study design and the use of case definitions. The remaining 8 studies were used for a meta-analysis [11-18]. See **Figure 1** for the PRISMA diagram on study selection.

The included papers were critically appraised using the modified Joanna Briggs Institute (JBI) Critical Appraisal checklist for prevalence studies [5,19] (Table S2; Appendix S2 in the **Online Supplementary Document**). For each eligible study, we extracted the following data: country where the study was conducted, urban/rural setting, the period of study, sample size (denominator), number of dementia cases (numerator) and/or unweighted dementia prevalence. Wherever possible, data by age group, gender and types of dementia were also extracted.

### Study characteristics

The 8 included studies took place in 2 countries of the SEAR – 7 from India [11-13,15-18] and 1 from Thailand [15]. Half of the studies were conducted in urban settings [11,16-18] while the other half were carried out in rural areas [12-15]. All studies took place between 2010 and 2017. Most studies adopted a two-stage design, which involved an initial screening by field workers and confirmation of dementia cases by a specialist. 2 out of 8 studies also included a third stage where researchers screen negative cases to identify false positives [11,15]. Most studies used a modified version of the Mini-Mental Status Exam (MMSE) as a screening tool. DSM-IV and ICD-10 were the most popular diagnostic tools used for case ascertainment. The study details can be found in **Table 1**.

**Table 1:** Study details

Study	Authors (Year)	Region/Country	Setting	Screening Tools	Outcome ascertainment	Study design
1	Banerjee (2017) [11]	Kolkata, India	Urban	KBSB	DSM-IV; NINCDS-ADRDA; NINCDS-AIREN	3-stage design cross-sectional study^*^
2	Gurukartick (2016) [12]	Thiruvennainallur in Villupuram District of Tamil Nadu, India	Rural	VSID	DSM-IV	2-stage design cross-sectional study†
3	Gambhir (2014) [13]	Chiraigaon block of Varanasi District, India	Rural	HMSE	DSM-IV-TR; ICD-10	2-stage design cross-sectional study†
4	Senanarong (2013) [14]	Siriraj, Thailand	Rural	TMSE	DSM-IV	2-stage design cross-sectional study†
5	Tiwari (2013) [15]	Luchnow, India	Rural	HMSE; CAMDEX-R	DSM-IV; ICD-10	3-stage design cross-sectional study*
6	Seby (2011) [16]	Pune district of Maharashtra State, India	Urban	GHQ-12; MMSE	ICD-10	2-stage design cross-sectional study†
7	Mathuranath (2010) [17]	Trivandrum, Kerala State, India	Urban	ACE; MMSE	DSM-IV; NINCDS-ADRDA; Hachinski’s Ischemic Scale	2-stage design cross-sectional study†
8	Saldanha (2010) [18]	Pune and Kirkee cantonments, Maharashtra, India	Urban	MMSE; CSI-D	ICD-10	Single phase cross-sectional survey

KCSB – Kolkata Cognitive Screening Battery, VSID – Vellore Screening Instrument for Dementia , HMSE – Hindi Mini Mental state examination, TMSE – Thai Mental State Examination, CAMDEX-R - Cambridge Examination for Mental Disorders of the Elderly - Revised (CAMDEX-R), GHQ-12 – General Health Questionnaire-12, MMSE – Mini Mental State Examination, ACE – Addenbrooke’s Cognition Examination, CSI-D – 10/66 Research Group Community Screening Instrument for Dementia, ICD – International Classification of Diseases, DSM – Diagnostic and Statistical Manual of Mental Disorders, NINDS-AIREN – National Institute of Neurological Disorders and Stroke Association criteria for vascular dementia, NINCDS-ADRDA – National Institute of Neurological and Communicative Disorders and Stroke and the Alzheimer’s Disease and Related Disorders Association criteria for Alzheimer’s disease

*3-stage design cross-sectional study: 1. Screening by trained fieldworkers; 2. Confirmation of suspected cases by consultant psychiatrists/ psychiatric team; 3. Checking of unsuspected cases by consult psychiatrists/ psychiatric team for false negatives.

†2-stage design cross-sectional study: 1. Screening by trained fieldworkers; 2. Confirmation of suspected cases by consultant psychiatrists/ psychiatric team.

The total sample size for the 8 studies combined was 28 543 participants. There was considerable variation in the number of participants between studies, ranging from 202 to 17 584 (**Table 2**). The median study sample size was 2072. All studies included individuals aged 50 years and above, and the most common classifications of age groups were age 60 years and above or age 65 years and above. Two studies recruited a regionally representative sample by taking into account the cultural and socioeconomic background of the participants [11-17]. The remaining studies used representative samples of the rural or urban community dwelling elderly populations. The proportion of female participants was around the same as male participants.

**Table 2:** Detailed sampling characteristics

Study	Authors (year)	Sample selection	Participant recruitment	Sample size and response rate	Participants traits
1	Banerjee (2017) [11]	Representative of the region in terms of socioeconomic and cultural levels	Stratified and random sampling	100 802 approached and analysed	47.2% female, ≥50 years old
				Attrition <1%	
2	Gurukartick (2016) [12]	Rural community dwelling elderly population	Random and proportional sampling	1304 analysed	44.9% female, ≥65 years old
				Sample size calculation ≥1300	
3	Gambhir (2014) [13]	Rural community dwelling elderly population	Random sampling	728 analysed	64.4% female, ≥60 years old
				54-80% for female	
4	Senanorong (2013) [14]	Rural community dwelling elderly population	Catchment from primary care unit of Siriraj Hospital	1998 approached, 1973 analysed (98.7%)	65.1% female, ≥60 years old
				Sample size calculation ≥1948	
5	Tiwari (2013) [15]	Rural community dwelling elderly population	Random sampling	2324 approached, 2146 analysed (92.3%)	52.6% female, ≥60 years old
				Sample size calculation ≥ 2060	
6	Seby (2011) [16]	Urban community dwelling elderly population	Consecutive sampling	218 approached, 202 analysed (92.7%)	49.1% female, ≥65 years old
7	Mathuranath (2010) [17]	Representative of the region in terms of socioeconomic and cultural levels	Door to door survey	2690 eligible, 2446 analysed (90.9%)	59.4% female, ≥55 years old
8	Saldanha (2010) [18]	Community dwelling population	Random sampling then door to door survey	2145 approached, 2119 analysed, (98.8%)	60.5% female, ≥65 years old

#### Quality assessment

There was considerable variation in the quality of the included studies (Appendix S2 in the **Online Supplementary Document**). The quality score ranged from 12 to 17 out of 18. Out of 8 studies, 2 study samples were representative of the target population [11,17] and 3 were sampled in an unbiased manner [11,14,17]. In general, sample sizes were appropriate, and 3 studies carried out a sample size calculation prior to recruitment [12,14,15]. Most studies clearly documented exclusion criteria, the number of refusals and loss to follow-up. Response rates were high overall: 6 had a response rate over 90% [11,14-18]. All studies used a well-recognized diagnostic manual and conducted different tests to exclude other conditions with similar clinical manifestations. For instance, all studies made effort to exclude depression as a differential diagnosis.

#### Data analysis

Given the paucity of data, dementia prevalence was estimated using a Bayesian approach and confirmed by a frequentist approach. This allows for comparison of the validity of estimates particularly given the limitations in data availability and the opportunity to explore the utility of using a simpler statistical package to generate a normal-normal hierarchical model (NNHM) for disease burden estimates with limited data. All analyses were conducted using the statistical software R (version 3.5.2, R Core Team, Vienna, Austria).

#### Bayesian approach

A Bayesian approach allows for estimates based on smaller numbers of studies by allowing prior estimates to be incorporated, which improves precision [7-9]. For instance, the 2009 estimate of 6.38% dementia prevalence in SEAR for people age ≥60 [4] indicates that the 2020 prevalence will probably be under 10%. If an updated meta-analysis included studies that were methodologically flawed or were based on a highly biased sample that produced an unrealistic prevalence (eg, >25%), the outlier effect would be restrained due to prior knowledge [9].

The interpretation of results generated by a Bayesian approach is also more intuitive. In the frequentist analysis, the concept of confidence interval is commonly misunderstood [20]. A 95% confidence interval (CI) means that if we were to take repeated samples from the population, and calculate the confidence intervals each time, then it would be expected that 95% of the calculated confidence intervals would be such as to include the true population parameter [21]. 95% credible interval produced from the Bayesian analysis, on the other hand, means that given the observed data, there is a 95% probability that the true value of prevalence falls within the credible interval [20]. This means that the credible intervals produced are much more relevant in global health research.

The age-specific prevalence was pooled using the bayesmeta package of R [20]. The Bayesian method can allow us to update the current state of knowledge by considering the newly extracted data alongside previously published literature. In 2009, Alzheimer's Disease International (ADI) published the age-specific prevalence of dementia for different world regions [4]. The age-specific prevalence of dementia in South Asia published by ADI was used to inform the specification of the prior for the Bayesian approach. Importantly, the ADI’s analysis was based on studies that are entirely distinct from those included in this work. Only studies with age-specific prevalence data were included in the meta-analysis. A Bayesian normal-normal hierarchical model (NNHM) was constructed with age groups as the independent variable and age-specific prevalence as the dependent variable. The NNHM model was chosen because it mirrors many of the key distributional assumptions embedded within the frequentist random-effects model [20,22].

The number of participants screened and the number of PWD identified from each study were first sorted into 10-year age group bins. Participants over the age of 80 were all allocated into an “over 80” bin. Previous research has held that dementia is rare among younger individuals and the prevalence of the disease increases with age. However, the evidence on the rate itself is mixed, particularly for older cohorts. To reflect this pattern of uncertainty, we set the prior variance to increase with age: for age groups 60-69, 70-79 and over 80, it was 0.092, 0.152 and 0.32 respectively. The prevalence estimates for each of these groups were pooled, and 95% credible intervals were obtained.

As a sensitivity test for the baysmeta package, Just another Gibbs sampler (JAGS) was used to construct a more traditional model simulated via Markov chain Monte Carlo (MCMC) methods, which produced estimates of disease burden for each age group bin highlighted above using a similar NNHM model. JAGS is an open source algorithm used often in Bayesian analysis to simulate draws from target posterior distributions [23].

#### Frequentist approach

Crude prevalence estimates were pooled using a frequentist approach with the metafor package of R [24]. Given the available evidence, which strongly indicates a degree of heterogeneity in prevalence rates within the region, a random effects model was thought most appropriate. Note the subtle point that we are interested in conducting inference about the SEAR dementia prevalence rate in general – that is, unconditional inference in the language of [25]. Again, this suggests the REM is the most suitable frequentist model to adopt. All studies with crude prevalence estimates were included in the analysis.

To assess heterogeneity between studies, Cochrane’s Q test, I-squared (I^2^) statistics and tau-squared (т^2^) statistics were examined. Cochrane’s Q test tests the null hypothesis that the true prevalence is the same in all primary studies included in the meta-analysis. A p-value of less than 0.05 shows evidence to reject the null hypothesis and indicates the presence of statistical heterogeneity among the included studies [26]. The I^2^ statistic represents the percentage of total variation across studies that is due to true heterogeneity instead of chance. Generally, I^2^ values of 25%, 50% and 75% are interpreted as low, moderate and high heterogeneity [27]. т^2^ is the variance of the prevalence parameter across the population of studies and reflects, again, heterogeneity in the true prevalence rate [28].

#### Burden estimation

The number of PWD in 2015, 2020 and 2030 was estimated by multiplying the prevalence obtained by both methods with the number of people from SEAR in 2015, 2020 and 2030 using data from the UN Population Division [29]. The burden of dementia in each age group was only estimated with the Bayesian model and is calculated by multiplying the age-specific prevalence and the number of people in the corresponding age groups in 2015, 2020 and 2030. Note these projections are made on a ceteris paribus basis: they allow only for changes to the population size; the prevalence rate and other key parameters are assumed to be constant through time.

## RESULTS

### Prevalence estimates

Five studies reported age-specific prevalence of dementia [12,14,17-19] and were included in the Bayesian NNHM. For people aged 60 and above, dementia prevalence was found to be 3.1% (95% credible interval = 1.5-5.0%). The prevalence of dementia ranged from 1.6% (95% credible interval = 0.8-2.5%) in those between 60-69 years of age to 12.4% (95% credible interval = 5.6-20%) in those above the age of 80 (**Table 3**). As a test of the sensitivity of the results, we conducted the analysis using a modified NNHM prior setup and estimated the model via classical MCMC methods: with that, we obtained similar results (Appendix S3 in the **Online Supplementary Document**).

**Table 3:** Prevalence of dementia in 10-year age groups in SEAR

Age	Pooled prevalence estimate (95% credible interval)	Number of PWD in SEAR in 2015 (thousands)	Projected number of PWD in SEAR in 2020 (thousands)	Projected number of PWD in SEAR in 2030 (thousands)
**60-69**	0.016 (0.008-0.025)	1691.90 (845.95-2,643.60)	2063.18 (1031.59-3223.73)	2733.55 (1366.78-4271.18)
**70-79**	0.034 (0.017-0.055)	1739.41 (869.70-2,813.75)	2,021.50 (1010.75-3270.08)	3115.76 (1557.88-5040.20)
**≥80**	0.124 (0.056-0.200)	2082.46 (940.46-3,358.80)	2579.70 (1165.02-4160.80)	3747.65 (1692.49-6044.60)
**≥60**	0.0314 (0.015-0.050)	5513.77 (2656.12-8816.15)	6664.38 (3207.37-10 654.61)	9596.96 (4,617.14-15 355.98)

PWD – people with dementia, SEAR – Southeast Asia Region

**Figure 2:**
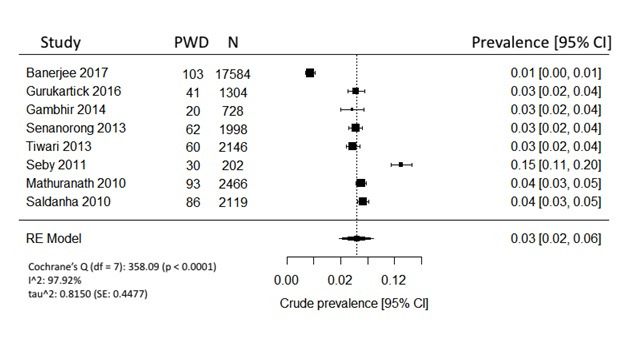
Crude prevalence for individuals over age 60.

All 8 studies reported overall dementia prevalence and were pooled using REM (**Figure 2**). Based on the frequentist model, the unadjusted crude prevalence for people over 60 years was estimated to be 3.0% (95% CI = 2-6%). Cochrane’s Q test revealed that there was significant heterogeneity among the included studies. True heterogeneity, indicated by I^2^, was above 90%, which was very high. The variance of prevalence parameter across the study populations, or т^2^, was 0.82.

### Burden estimates

**Figure 3:**
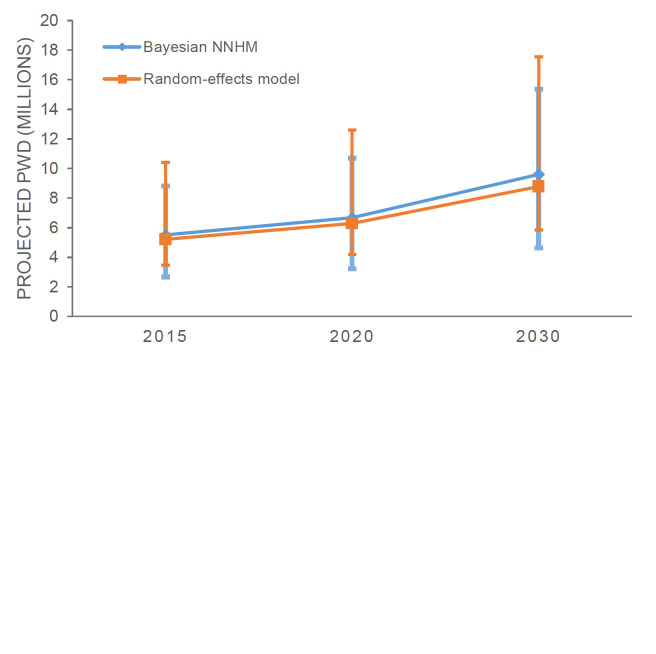
Projected dementia cases in SEAR by Bayesian and frequentist models. Note: Due to the conceptual differences between the analytic approaches we would like to remind the reader that the 95% confidence intervals (Random-effects model) and 95% credible intervals (Bayesian) cannot be interpreted interchangeably.

Overall, there were 175.7 million people aged 60 and above in SEAR in 2015. This number is projected to increase to 209.2 million in 2020 and 292.7 million in 2030 [29]. Based on the results obtained with the Bayesian NNHM model, we estimate that the number of PWD will increase from 5.51 million (95% credible interval = 2.66-8.82 million) in 2015 to 6.66 million (95% credible interval = 3.21-10.7 million) in 2020. In 2030, it is projected that there will be nearly 10 million (9.60 million, 95% credible interval = 4.62-15.36 million) PWD in SEAR (**Figure 3**, **Table 4**). Similarly, based on the REM, we estimate there were 5.21 million (95% CI = 3.47-10.40 million) PWD in SEAR in 2015 (**Table 4**). The number of PWD is projected to increase to 6.28 million (95% CI = 4.18-12.6 million) in 2020 and 8.78 million (95% CI = 5.85-17.60 million) in 2030.

**Table 4:** Burden estimate comparisons between Bayesian and frequentist models

Year	Bayesian NNHM	Random-effects model
**2015**	5.51 (2.66-8.82) million*	5.21 (3.47 - 10.40) million
**2020**	6.66 (3.21-10.7) million	6.28 (4.18 – 12.60) million
**2030**	9.60 (4.62-15.36) million	8.78 (5.85 –17.56) million

NNHM – normal-normal hierarchical model

The Bayesian NNHM model was also used to estimate the burden of dementia in each 10-year age group (**Table 3**). People over the age of 80 had the highest estimated burden of dementia, with 2.08 million (95% credible interval = 0.94-3.36 million) in 2015. This burden is projected to rapidly increase to 2.58 million (95% credible interval = 1.17-4.16 million) in 2020 and 3.75 million (95% credible interval = 1.69-6.04 million) in 2030.

## DISCUSSION

We aimed to estimate the prevalence of dementia in the WHO’s SEAR and adopted both Bayesian and traditional frequentist approaches to optimize our understanding of the burden of dementia within this region. We searched for studies published in the English language between 2010 and 2018 and ultimately found 8 for inclusion, 7 of which were from India, and 1 was conducted in Thailand. The Bayesian approach may be more useful in regions with limited data availability. We therefore compared it to the traditional frequentist approach, which is more commonly used in studies with larger amounts of data available. Our Bayesian estimates revealed that there were about 5.51 million people living with dementia in SEAR in 2015, consistent with the most recent estimates produced by IHME, which found that there were 5.47 million people with dementia in SEAR in 2016 [6].

Despite our best efforts to estimate dementia prevalence in Southeast Asia, current epidemiological knowledge of dementia prevalence in this region proved to be sparse. Out of the eleven countries in the area, only India and Thailand provided studies with sufficient data. Indonesia and Bangladesh are the third most populated countries in Southeast Asia after India, which may be most affected by dementia in the upcoming years, yet lack of published studies makes it extremely difficult to assess and predict the impact dementia will have on these countries. With an estimated 5.51 million already living with dementia and close to 10 million projected to be affected by 2030, urgent public health action is necessary.

The crude prevalence of dementia in people aged 60 years and above was 3.0% (95% CI = 2-6%) based on all included studies. The age-adjusted prevalence of dementia was 3.1% (95% credible interval = 1.5-5.0%). As the majority of the included studies in this current review came from India, the most comparable (external) prevalence estimate was an estimate produced by the Alzheimer’s and Related Disorders Society of India in 2010. They found that the prevalence of dementia in India ranged from 0.6% to 3.5% in rural areas and 0.9% to 4.8% in urban areas [30]. Our estimate, therefore, is consistent with the findings of current literature. This review also supports the long established relationship between age and prevalence of dementia. The prevalence ranged from 1.6% in the 60-69 age group to 12.4% in the above 80 age group. In comparison, sensitivity analysis using Bayesian approach with the JAGS sampler showed similar though slightly higher prevalence estimates with 2.3% for ages 60-69, 4.9% for ages 70-79, 13.5% for above age 80, respectively.

The Delphi Consensus reported prevalence of dementia for two regions in Southeast Asia – SEAR B which includes Indonesia, Thailand and Sri Lanka, and SEAR D which is comprised of India and South Asian countries. Their estimates for SEAR D which include India and are most comparable to this research show that the prevalence of dementia varied between 0.4% in those between 60-64 to 14% in people above 85. However, it is more difficult to make comparisons of current estimates to the SEAR B region, as this review did not include any study from those regions in the data analysis. A more recent meta-analysis conducted by Prince et al in 2013 included five studies that showed how dementia prevalence in the South Asia region (most comparable to this review as it included India) for individuals over age 60 was 5.8% [5].

Given that an optimal approach to estimating disease burden continues to be an area of research, the results lend validity to the use of the Bayesian NNHM approach as adopted in this review and support its further use. Future research is needed to explore the role of varying statistical approaches in prevalence and burden of disease estimates, but the results of this paper are promising, particularly in settings with limited data availability as in this review. Furthermore, the ability to use a user-friendly open source software to run complex full Bayesian analysis like bayesmeta allows for easier incorporation of this approach in future studies and allows greater accessibility to estimate burden of disease for researchers across the globe.

The use of PRISMA guidelines, careful selection of studies, and the use of validated quality assessment tools to ensure research rigour were strengths of this review. However, despite efforts to provide the best possible estimate of the prevalence of dementia in SEAR, there were some limitations. First, while the studies were carefully selected and chosen based on the quality of conducted research, the sample size of each study was relatively small compared to the overall population of the respective countries. Second, the estimates of the current study were based on only 2 of the 11 countries in the SEAR (mainly India, and to a lesser extent Thailand). The paucity of data from the rest of the region represents a serious limitation of our work. Third, the other countries in this region all have very distinct cultures and levels of development, which would plausibly lead to variation in their citizens’ level of dementia risk. Fourth, the articles were restricted to English language only, potentially limiting the research from countries which may not have a large number of English publications. Fifth, some studies only included participants that speak Hindi, Thai or English, possibly underestimating the true number of participants suffering from dementia as many may be excluded for not speaking these languages. Sixth, sex-and age-specific prevalence of dementia could not be estimated due to lack of reporting from the included studies. Seventh, very few studies reported information on specific dementia types, making it difficult to estimate the prevalence of dementia subtypes (eg, Alzheimer’s disease, vascular dementia). Lastly, it is worth underscoring that our estimates of the burden of dementia crucially relied on a number of strong assumptions: the prevalence remains the same and the population structure progresses as predicted by the UN estimates. It is likely that these will not be borne out. Nevertheless, the estimates give researchers and policymakers important and relevant insight into the future trend of dementia burden.

Dementia has been significantly underrecognized and underestimated in SEAR. This review estimated the prevalence of dementia in SEAR and showed that the number of people living with dementia in this region is substantial and is expected to rise, with over 10 million people projected to have dementia in this region by 2030. In 2015, the total costs of dementia in Southeast Asia reached US$7.3 billion, which was around one third of Nepal’s national GDP [1,31]. With the projected increase in dementia prevalence, the costs incurred will place a significant financial toll on governments, families and individuals.

There is an urgent need for more epidemiological research on dementia burden in SEAR. Nine out of eleven countries in SEAR have no published information on the prevalence of dementia. For future epidemiological studies, there should be greater emphasis on reporting of research findings. Apart from the crude dementia prevalence, all studies should aim to report on age-specific and sex-and-age-specific dementia prevalence. If resources allow, researchers should also attempt to estimate the prevalence of dementia subtypes. This information will be very useful for more accurate burden estimation and healthcare planning.

## CONCLUSION

This review calls for greater recognition of dementia as a health priority and urges countries in SEAR to reassess their approach to addressing dementia. We found a significant burden of dementia in SEAR that is projected to nearly double by 2030. Governments should focus on promoting awareness of the condition and giving recognition to the fact that dementia is not just a by-product of aging, but a recognized illness that has broad effects on the healthcare system and economy.

We demonstrated that the Bayesian approach offers benefits beyond the traditional frequentist approach to estimate disease prevalence and burden, particularly with significantly limited data. We were also able to demonstrate that the bayesmeta package running an NNHM model can offer similar estimates to the Bayesian JAGS algorithm. Further research is needed to assess if there is an optimal approach for disease burden estimates. The Bayesian approach offers a promising methodology for improved estimates and should continue to be explored, especially since accessible open source software for this method is now available.

Future research should focus on targeting risk factors for dementia in SEAR as well as possible prevention measures. These future directions will help address the needs of and bring tailored interventions to various types of communities affected by dementia in these countries, with the long-term goal of generating greater understanding and policies to decrease the prevalence of this disabling disorder.

## Additional material

Online Supplementary Document
