# Supplementary material for: Systematic review estimating the burden of dementia in the WHO Southeast Asia Region using Bayesian and frequentist approaches
Source: J Glob Health. 2020 Sep 23;10(2):020701. doi: 10.7189/jogh.10.020701 (PMC7688200; doi:10.7189/jogh.10.020701)
Supplement: Online Supplementary Document [file jogh-10-020701-s001.pdf]

# Online Supplementary Document

**APPENDIX 1.** Search Terms

The search terms were (“dementia”[Mesh] OR “Alzheimer”[Mesh]) AND (“prevalence”[Mesh] OR “incidence”[Mesh] OR “morbidity”[Mesh] OR “epidemiology”[Mesh] OR “burden of disease”[Mesh] OR “mortality”[Mesh]) AND (Southeast Asia OR Indonesia OR Sri Lanka OR Thailand OR Timor-Leste OR Bangladesh OR Bhutan OR India OR Korea OR "Democratic People’s Republic of Korea" OR "Indian ocean islands" OR Maldives OR Myanmar OR Nepal).

**Table S1.** Search terms used in varying databases.

| Database | Search details |
|---|---|
| **MEDLINE** (May 10, 2018) | 1. exp PREVALENCE/ 252216<br>2. exp MORBIDITY/ 465368<br>3. exp EPIDEMIOLOGY 24821<br>4. exp MORTALITY 341992<br>5. exp INCIDENCE 229870<br>6. ‘Global Burden of Disease’/ 132<br>7. (Prevalen* or inciden*).mp 1313350<br>8. (disease adj3 burden).mp 18000<br>9. 1 or 2 or 3 or 4 or 5 or 6 or 7 or 8 1647359<br>10. dementia/or dementia, vascular/or dementia, multi-infarct/or frontotemporal lobar degeneration/or frontotemporal dementia/or Lewy body disease/ 54286<br>11. Alzheimer Disease/ 82809<br>12. Alzheimer*.mp 120500<br>13. 10 or 11 or 12 158294<br>14. Asia, Southeastern/ 7353<br>15. Indonesia/ 8815<br>16. Sri Lanka/ 5207<br>17. Thailand/ 23621<br>18. Timor-Leste/ 149<br>19. Bangladesh/ 9091<br>20. Bhutan/ 346<br>21. India/ 91472<br>22. Korea/or ‘Democratic People’s Republic of Korea’ 16455<br>23. Indian Ocean Islands/ 705<br>24. Maldives.mp 204<br>25. Myanmar/ 1908<br>26. Nepal/ 6625<br>27. 14 or 15 or 16 or 17 or 18 or 19 or 20 or 21 or 22 or 23 or 24 or 25 or 26 166917<br>28. 9 and 13 and 27 125 |

| | |
|---|---|
| | 29. limit 28 to (humans and yr='2010-Current') 54 |
| **PUBMED** (MAY 10, 2018) | Search (((dementia OR Alzheimer* OR Alzheimer's disease))AND(prevalence OR incidence OR morbidity OR mortality OR epidemiology OR burden OR rate)) AND (Southeast Asia OR Thailand OR Thai* OR Timor Leste OR Indonesia OR India OR Maldives OR Nepal OR Democratic People's Republic of Korea OR Bangladesh OR Bhutan OR Myanmar OR Sri Lanka):<br>**RESULTS: 470** |
| **EMBASE** (May 10, 2018) | 1. exp PREVALENCE/ 612183<br>2. exp MORBIDITY/ 318050<br>3. exp EPIDEMIOLOGY 28448523<br>4. exp MORTALITY 926000<br>5. (Prevalen* or inciden*).mp 2032187<br>6. 1 or 2 or 3 or 4 or 5 3731549<br>7. Alzheimer Disease/ 174594<br>8. Alzheimer*.mp 212419<br>9. exp dementia/ 314248<br>10. 7 or 8 or 9 343859<br>11. Southeast Asia/ 9186<br>12. Indonesia/ 13622<br>13. Sri Lanka/ 7089<br>14. Thailand/ 29349<br>15. Timor-Leste/ 437<br>16. Bangladesh/ 13562<br>17. Bhutan/ 583<br>18. North Korea/or Korea 34431<br>19. India/ 125778<br>20. Maldives/ 216<br>21. Myanmar/ 2812<br>22. Nepal/ 9508<br>23. 11 or 12 or 13 or 14 or 15 or 16 or 17 or 18 or 19 or 20 or 21 or 22 234393<br>24. 6 and 10 and 23 571<br>25. limit 28 to (humans and yr='2010-Current') 379 |
| **PSYCINFO** (MAY 10, 2018) | 1. exp Epidemiology 43822<br>2. Prevalence.mp. 99900<br>3. exp Morbidity 5010<br>4. mortality.mp. 35279<br>5. incidence.mp. 38569<br>6. (Prevalen* or inciden*).mp. 177883<br>7. (burden adj3 disease).mp. 3267<br>8. 1 or 2 or 3 or 4 or 5 or 6 or 7 224194 |

| | |
|---|---|
| | 9. dementia/or dementia with lewy bodies/or senile dementia/or vascular dementia 33731<br>10. exp Alzheimer Disease/ 41225<br>11. Alzheimer*.mp 55801<br>12. 9 or 10 or 11 75805<br>13. Indonesia.mp. 1939<br>14. Sri Lanka.mp. 1121<br>15. Thailand.mp. 3160<br>16. Timor-Leste.mp. 63<br>17. Bangladesh.mp. 1449<br>18. Bhutan.mp. 134<br>19. India.mp. 14192<br>20. Maldives.mp. 44<br>21. Myanmar.mp. 217<br>22. Nepal.mp. 1106<br>23. North Korea.mp. 118<br>24. Southeast asia.mp. 826<br>25. 13 or 14 or 15 or 16 or 17 or 18 or 19 or 20 or 21 or 22 or 23 or 24 22876<br>26. 8 and 12 and 25 74<br>27. limit 26 to (humans and yr='2010-Current') 36 |
| **GLOBAL HEALTH LIBRARY** (MAY 10, 2018) | 1. incidence/or disease incidence 97442<br>2. (prevalen* or inciden*).mp. 465707<br>3. epidemiology/or disease prevalence/or morbidity/or mortality 415286<br>4. (burden adj3 disease).mp. 9648<br>5. 1 or 2 or 3 or 4 667109<br>6. dementia/ 7694<br>7. alzheimer disease/ 7211<br>8. Alzheimer*.mp 9311<br>9. 6 or 7 or 8 14473<br>10. south east asia/ 92988<br>11. Indonesia 12080<br>12. Sri Lanka 6038<br>13. Thailand/ 21377<br>14. Timor-Leste/ 285<br>15. Bangladesh/ 10246<br>16. Bhutan/ 410<br>17. North Korea.mp.or Korea Democratic Peoples Republic/ 318<br>18. India/ 111063<br>19. Maldives/ 178<br>20. Nepal 5657<br>21. Myanmar 3463<br>22. 10 or 11 or 12 or 13 or 14 or 15 or 16 or 17 or 18 or 19 or 20 or 21 218966<br>23. 5 and 9 and 22 180 |

| | |
|---|---|
| | 24. limit 23 to (humans and yr='2010-Current') 138 |
| **WEB OF SCIENCE** (MAY 10, 2018) | TOPIC: (dementia OR Alzheimer's disease OR Alzheimer*) AND TOPIC: (prevalence OR incidence OR epidemiology OR burden OR morbidity OR mortality) AND TOPIC: (southeast Asia OR Indonesia OR Sri Lanka OR Thailand OR Timor Leste OR India OR Maldives OR Myanmar OR Nepal OR Democratic People s Republic of Korea OR North Korea OR Bhutan OR Bangladesh) **RESULTS: 185** |
| **BIOSIS** (MAY 10, 2018) | TOPIC: (dementia OR Alzheimer's disease OR Alzheimer*) AND TOPIC: (prevalence OR incidence OR epidemiology OR burden OR morbidity OR mortality) AND TOPIC: (southeast Asia OR Indonesia OR Sri Lanka OR Thailand OR Timor Leste OR India OR Maldives OR Myanmar OR Nepal OR Democratic People s Republic of Korea OR North Korea OR Bhutan OR Bangladesh) **RESULTS: 68** |
| **GLOBAL INDEX MEDICUS** (MAY 10, 2018) | Search: Dementia **RESULTS: 226** |

***Appendix 2. Quality Assessment***

The included papers were critically appraised with the aid of Joanna Briggs Institute (JBI) Critical Appraisal checklist for prevalence studies (104). The JBI checklist was modified based on Prince et al. to suit this review (60). The original JBI checklist contained 9 items that could be answered with yes or no. For this review, the checklist was converted into a scale. A score of 2 (Excellent), 1 (Good) or 0 (Not available/ Unclear/ Poor) was assigned to each item, and the score was summed across items to generate an overall quality score that ranged from 0 to 18.

***Quality Assessment***

The modified JBI checklist (Table 3) was a helpful tool for identifying selection bias and information bias. Out of the 9 items assessed, 6 (item 1-5 and 9) looked at selection bias, 2 (item 6 and 7) focused on information bias and 1 (item 8) examined statistical analysis method for reducing bias. There was considerable variation in the quality of the included studies, ranging from 12 to 17 out of 18.

The following aspects of selection bias were assessed: sample population, sampling method, sample size, study subject and setting description, sufficient coverage (i.e. details about missing data) and response rate. Out of 8 studies, 2 study samples were representative of the target population; and 3 samples were sampled in an unbiased manner (ref). In general, sample sizes were good, 5 studies had a sample size larger than 150 and 3 studies carried out a sample size calculation prior to recruitment. A sufficient sample size can lead to more precise estimate. Most studies clearly documented exclusion criteria, number of refusals and loss to follow-up. Response rates were high overall: apart from two studies, all studies included had a response rate over 90%.

All studies demonstrated a commendable effort at reducing information bias. All studies used a well-recognised diagnostic manual and conducted different tests to exclude other conditions with similar clinical manifestations. For instance, all studies made an effort to exclude depression as a differential diagnosis. Outcome measurements were conducted in a standardised and reliable way for most of the studies (7 out of 8).

All studies only presented the crude and unadjusted prevalence of dementia. Given that some studies did not have a representative sample that correspond to the population structure of the population, the unadjusted prevalence may not reflect the true prevalence of the target population.

**JBI Critical Appraisal Checklist for Studies Reporting Prevalence Data**

Reviewer Date

Author Year______________Record Number

| | 2 | 1 | 0 |
|---|---|---|---|
| 1. Was the sample frame appropriate to address the target population? | □ | □ | □ |
| 2. Were study participants sampled in an appropriate way? | □ | □ | □ |
| 3. Was the sample size adequate? | □ | □ | □ |
| 4. Were the study subjects and the setting described in detail? | □ | □ | □ |
| 5. Was the data analysis conducted with sufficient coverage of the identified sample? | □ | □ | □ |
| 6. Were valid methods used for the identification of the condition? | □ | □ | □ |
| 7. Was the condition measured in a standard, reliable way for all participants? | □ | □ | □ |
| 8. Was there appropriate statistical analysis? | □ | □ | □ |
| 9. Was the response rate adequate, and if not, was the low response rate managed appropriately? | □ | □ | □ |

Overall appraisal: Include □Exclude □Seek further info □
Comments (Including reason for exclusion)

1. **Was the sample frame appropriate to address the target population?**

   2 Nationally/ regionally representative sample

   1 Locally representative (limited generalisability)

   0 Unrepresentative of any population

2. **Were study participants recruited in an appropriate way?**

   2 Random sampling AND detailed recruitment process

   1 Consecutive sampling OR (random sampling AND unclear recruitment process)

   0 Unspecified

3. **Was the sample size adequate?**

   2 >1500

   1 500-1500

   0 <500

4. **Were the study subjects and setting described in detail?**

   2 Detailed description

   1 Limited description/ Unclear description

   0 No description

5. **Was data analysis conducted with sufficient coverage of the identified sample?**

   2 Detailed description of refusal to participate, loss to follow up and exclusion criteria

   1 Limited description of refusal to participate, loss to follow up and exclusion criteria

   0 Unspecified/ Unclear description

6. **Were valid methods used for the identification of the condition?**

   (The use of diagnostic manual was part of inclusion criteria for this review)

   2 Use of well recognised diagnostic manual AND exclusion of differential diagnoses (e.g. depression)

   1 Use of well recognised diagnostic manual

   0 No use of diagnostic manual

7. **Was the condition measured in a standard, reliable way for all participants?**

2 The condition was measured in a standard AND reliable way for all participants

1 The condition was measured in a reliable way for all participants

0 The condition was not measured properly

8. **Was there appropriate statistical analysis?**

2 Both crude and adjusted (or weighted) prevalence were calculated

1 Only crude prevalence was calculated

0 Statistical analysis was not conducted properly

9. **Was the response rate adequate, and if not, was the low response rate managed appropriately?**

2 ≥80%

1 60-79%

0 <60%

**Table S2. Quality assessment based on modified Joanna Briggs Institute (JBI) Critical Appraisal checklist**

| Item of JBI checklist | | 1 | 2 | 3 | 4 | 5 | 6 | 7 | 8 |
|---|---|---|---|---|---|---|---|---|---|
| Study | **Authors (Year)** | **Sample frame** | **Sampling method** | **Sample size** | **Study subject and setting description** | **Sufficient coverage** | **Outcome ascertainment** | **Outcome measurement** | **Statistical analysis** |
| 1 | Banerjee (2017) | 2 | 2 | 2 | 2 | 1 | 2 | 2 | 1 |
| 2 | Gurukartick (2016) | 1 | 2 | 1 | 2 | 1 | 2 | 2 | 1 |
| 3 | Gambhir (2014) | 1 | 1 | 1 | 2 | 0 | 2 | 2 | 1 |
| 4 | Senanorong (2013) | 1 | 1 | 2 | 2 | 2 | 2 | 2 | 1 |
| 5 | Tiwari (2013) | 1 | 1 | 2 | 2 | 2 | 2 | 2 | 1 |
| 6 | Seby (2011) | 1 | 1 | 0 | 2 | 2 | 2 | 1 | 1 |
| 7 | Mathuranath (2010) | 2 | 2 | 2 | 2 | 2 | 2 | 2 | 1 |
| 8 | Saldanha (2010) | 1 | 1 | 2 | 2 | 2 | 2 | 2 | 1 |

**-Each item was assigned 2 marks: 2-Excellent; 1-Good; 0-Not available/ Unclear/ Poor**
**-The maximum quality score achievable was 18 (2 X 9 items)**
**-Refer to Appendix 2 for specific details**

**Appendix 3. Sensitivity test for Bayesian Normal-Normal Hierarchal Model**

| Age | Traditional Bayesian with JAG algorithm | | Bayesian NNHM with bayesmeta | |
|---|---|---|---|---|
| | Mean | Standard Deviation | Mean | 95% Credible Interval |
| 60-69 | 0.0230 | 0.019 | 0.016 | (0.008-0.025) |
| 70-79 | 0.0491 | 0.046 | 0.034 | (0.008-0.025) |
| 80-89 | 0.135 | 0.096 | 0.124 | (0.056-0.200) |
